# Exploiting DNA Damage Repair in Precision Cancer Therapy: BRCA1 as a Prime Therapeutic Target

**DOI:** 10.3390/cancers13143438

**Published:** 2021-07-09

**Authors:** Liliana Raimundo, Juliana Calheiros, Lucília Saraiva

**Affiliations:** LAQV/REQUIMTE, Laboratόrio de Microbiologia, Departamento de Ciências Biolόgicas, Faculdade de Farmácia, Universidade do Porto, 4050-313 Porto, Portugal; liliana-raimundo@live.com (L.R.); up202010038@edu.ff.up.pt (J.C.)

**Keywords:** DNA damage repair, BRCA1, synthetic lethality, targeted anticancer therapy

## Abstract

**Simple Summary:**

Chemical inhibition of central DNA damage repair (DDR) proteins has become a promising approach in precision cancer therapy. In particular, BRCA1 and its DDR-associated proteins constitute important targets for developing DNA repair inhibiting drugs. This review provides relevant insights on DDR biology and pharmacology, aiming to boost the development of more effective DDR targeted therapies.

**Abstract:**

Precision medicine aims to identify specific molecular alterations, such as driver mutations, allowing tailored and effective anticancer therapies. Poly(ADP)-ribose polymerase inhibitors (PARPi) are the prototypical example of targeted therapy, exploiting the inability of cancer cells to repair DNA damage. Following the concept of synthetic lethality, PARPi have gained great relevance, particularly in BRCA1 dysfunctional cancer cells. In fact, BRCA1 mutations culminate in DNA repair defects that can render cancer cells more vulnerable to therapy. However, the efficacy of these drugs has been greatly affected by the occurrence of resistance due to multi-connected DNA repair pathways that may compensate for each other. Hence, the search for additional effective agents targeting DNA damage repair (DDR) is of crucial importance. In this context, BRCA1 has assumed a central role in developing drugs aimed at inhibiting DNA repair activity. Collectively, this review provides an in-depth understanding of the biology and regulatory mechanisms of DDR pathways, highlighting the potential of DDR-associated molecules, particularly BRCA1 and its interconnected partners, in precision cancer medicine. It also affords an overview about what we have achieved and a reflection on how much remains to be done in this field, further addressing encouraging clues for the advance of DDR targeted therapy.

## 1. Introduction

Cancer is a major burden of disease and one of the leading barriers to improve life expectancy worldwide [1]. Despite many efforts to maximize cancer prevention, diagnosis and treatment, the incidence and mortality rates have been steadily increasing [1,2].

As a highly heterogeneous disease, cancer displays unique genomic and epigenetic variations among patients. As such, the success of conventional chemotherapeutics has been limited by the heterogeneity of patients’ response, development of resistance and severity of side effects [3,4]. Conversely, targeted therapies take advantage of specific alterations in cancer cells, having emerged as a hopeful strategy to overcome these limitations [5,6,7,8].

There is a large amount of evidence that dysfunctional DNA damage repair (DDR) processes are frequently observed in cancer and are associated with genomic instability [5,7,9]. In fact, although cells own an equipped machinery to repair DNA toxic lesions, it may fail, predisposing them to accumulate DNA damage. The survival and proliferation of unrepaired DNA defective cells lead to the accumulation of mutations and genomic instability, strongly contributing to cancer development [5,10]. Nevertheless, cancer-associated DDR defects can also give rise to vulnerabilities that can be therapeutically exploited [5]. Indeed, DDR-deficient cells are frequently associated with hypersensitivity to DNA-damaging agents [11,12,13,14,15]. This evidence highlights the importance of the DDR regulatory molecules in targeted anticancer therapy.

Breast cancer susceptibility gene 1 (*BRCA1*) is a tumour suppressor gene extensively involved in maintaining genomic integrity through multiple functions in DDR, transcriptional regulation, cell cycle checkpoint and protein ubiquitination [16,17,18]. BRCA1 is frequently dysfunctional in human breast, ovarian, pancreatic, among other cancers, contributing to the accumulation of genomic defects. Also, *BRCA1* germline mutations account for most known heritable forms of cancer such as hereditary breast and ovarian cancer (HBOC) syndrome [19]. Despite the increased risk conferred by *BRCA1* mutations to cancer onset, pre-clinical and clinical data have ascertained that BRCA1 impairment is commonly associated with chemosensitivity in cancer cells [13,16]. BRCA1 has therefore become an important predictive and therapeutic molecule for developing targeted anticancer strategies. Other players involved in DDR can also be found defective in cancer, including breast cancer susceptibility gene 2 (*BRCA2*), *RAD51*, *RAD52*, partner and localizer of BRCA2 (*PALB2*), ataxia-telangiectasia mutated (*ATM*) and ataxia telangiectasia and Rad3-related (*ATR*), constituting additional encouraging targets for cancer treatment [6,20].

This review aims to emphasize the potentiality of the DDR pathways, particularly of BRCA1 and interconnected molecules, in precision cancer therapy.

## 2. DNA Damage Repair Pathways

**Figure 1:**
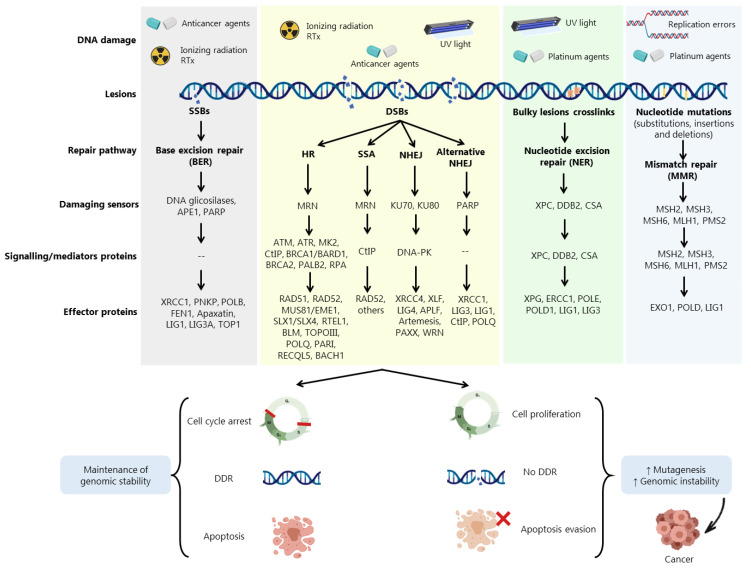
DNA damage agents and cellular repair pathways. DNA damage repair (DDR) pathways are activated in response to endogenous stresses (e.g., base depurination, deamination and reactive by-products of cellular metabolism) or exogenous exposure to different types of radiation or genotoxic agents. DDR comprises a network of proteins that are either DNA damage sensors, signalling mediators or effector proteins that execute DNA repair. The base excision repair (BER) pathway for single-strand breaks (SSBs), repairs minor DNA changes originated from oxidized or alkylated bases and small base adducts, with poly(ADP)-ribose polymerase (PARP) being the major player. The nucleotide base excision repair (NER) pathway deals with modified nucleotides that change the double helix structure, such as those induced by ultraviolet (UV) light. The mismatch repair (MMR) pathway deals with DNA damage that disturb the DNA helical structure and replication errors as substitution, insertions and deletions. Four different DDR mechanisms are described for double-strand breaks (DSBs) repair: homologous recombination (HR), non-homologous end joining (NHEJ), alternative NHEJ and single-strand annealing (SSA) pathways. Loss or aberrations in DDR proteins allows cell cycle proliferation and evasion of apoptotic events, resulting in increased genomic instability and cancer development. Radiotherapy (Rtx); Apurinic/apyrimidinic endonuclease 1 (APE1); MRE11/RAD50/NSB1 (MRN); Xeroderma pigmentosum, complementation group C (XPC); DNA damage-binding protein 2 (DDB2); Cockayne syndrome group A (CSA); MutS homolog 2, 3 and 6 (MSH2, MSH3 and MSH6); MutL homolog 1 (MLH1); Ataxia-telangiectasia mutated (ATM); Ataxia telangiectasia and Rad3-related (ATR); Mitogen-activated protein kinase-2 (MK2); *C*-terminal-binding interacting protein (CtIP); Breast cancer susceptibility 1 and 2 (BRCA1 and BRCA2); BRCA1-associated RING domain (BARD1); Partner and localizer of BRCA2 (PALB2); Replication protein A (RPA); DNA-dependent protein kinase (DNA-PK); X-Ray repair cross complementing 1 and 4 (XRCC1 and XRCC4); Polynucleotide kinase 3’-phosphatase (PNKP); DNA polymerase beta (POLB); Flap structure-specific endonuclease 1 (FEN1); DNA ligase 1, 3A and 4 (LIG1, LIG3A and LIG4); DNA topoisomerase 1 and 3 (TOP1 and TOPOIII); Essential meiotic structure-specific endonuclease 1 (EME1); Regulator of telomere elongation helicase 1 (RTEL1); Bloom syndrome protein (BLM); DNA polymerase theta (POLQ); PCNA-associated recombination inhibitor protein (PARI); RecQ like helicase 5 (RECQL5); BRCA1-associated *C*-terminal helicase (BACH1); XRCC4-like factor (XLF); Aprataxin and PNKP like factor (APLF); Werner syndrome helicase (WRN); Xeroderma pigmentosum group G (XPG); Excision repair cross-complementation group 1 (ERCC1); DNA polymerase epsilon (POLE); DNA polymerase Delta 1 (POLD1); Exonuclease 1 (EXO1); DNA polymerase delta 1 (POLD).

DDR is activated in response to different endogenous and exogenous stresses [5,21] (Figure 1). When aberrantly repaired, DNA damage might be associated with clinical outcomes such as neurodegeneration, infertility, and genomic instability, being a key contributing factor to neoplastic transformation and tumour development [8] (Figure 1). Due to the complexity underneath detection and repair of DNA damage, cells evolved an intricate DDR network that, together with cell cycle regulation, promotes the maintenance of genomic stability and cellular viability [22] (Figure 1).

Cells harbouring defects on a particular DDR pathway may compensate by becoming reliant on another repair pathway. In fact, despite showing partially overlapping functions, DDR pathways still exhibit different functionalities depending on multiple damage sensors, signalling factors (activators of cell cycle checkpoints) and effector DDR proteins (Figure 1). In Figure 1, the main pathways responsible for processing a distinct DNA damage, such as single-strand breaks (SSBs) and double-strand breaks (DSBs), are represented [5,8,21,22,23,24]. DSBs are among the most deleterious DNA lesions, leading to apoptosis when unrepaired. Conversely, misrepaired DSBs may generate mutations or chromosome rearrangements that may lead to a malignant condition [23]. Three main mechanisms are required for DSBs repair: (i) damage detection, (ii) ability to control cell cycle and transcriptional programs, and (iii) mechanisms for catalysing the repair of the lesion [23].

Accumulated data have shown the involvement of DDR proteins in different stages of cancer development. Early stages of tumorigenesis have been associated with activation of DDR proteins due to the induction of replication stress and DNA damage, acting as a barrier to the proliferation of aberrant cells [22]. However, most of pre-malignant cells are able to escape this barrier by loss or aberrations in specific proteins associated with DDR and cell cycle regulation, such as BRCA1, BRCA2, ATM, RAD51, Fanconi anemia group A protein (FANCA) and p53, allowing these cells to evolve to malignant carcinomas (Figure 1). In more advanced stages, when the tumour is already established, (re)activation and overexpression of DDR factors support cells to evade the lethal effect of the therapeutic agents, eliciting resistance [22].

## 3. Role of BRCA1 in DSBs Repair

In 1990, *BRCA1* (located on chromosome 17) was identified as a classical tumour suppressor gene (TSG) due to the loss of a wild-type (wt) allele during tumorigenesis, being the first TSG associated with hereditary and sporadic cases of basal-like breast cancer [25,26].

Despite being a multifunctional protein, the BRCA1 tumour suppressive function is mainly ensured by its ability to maintain genomic integrity through regulation of diverse cellular processes, including DDR, cell cycle checkpoint, apoptosis, chromosome instability, among others [16,17]. The BRCA1 effect on DDR seems to mainly occur through regulation of homologous recombination (HR) [17] (Figure 1). In fact, most mutant BRCA1 (BRCA1^Mut^) forms are defective in HR activity, although in varying grades depending on the location of the mutation [27]. Although poorly understood, BRCA1 may also participate in non-homologous end joining (NHEJ), alternative NHEJ, and single-strand annealing (SSA) repair pathways [8,28]. Upon DNA damage, the opposite roles played by p53-binding protein 1 (53BP1) and BRCA1 seem to support cells in the switch between NHEJ and HR [28]. However, this mechanism is not completely understood [28]. Studies have also revealed that BRCA1 interacts with Ku80 (a crucial protein in NHEJ), being recruited to DSBs sites in a Ku80-dependent manner [29]. In fact, DNA repair pathways compete to select which mechanism should be employed. This choice is based on several factors, including cell cycle phase. Somatic cells use error-prone NHEJ as a major DSBs repair mechanism throughout all cell cycle stages, but particularly occurring in G1 phase, while HR is employed predominantly in S to G2 phases [30].

**Figure 2:**
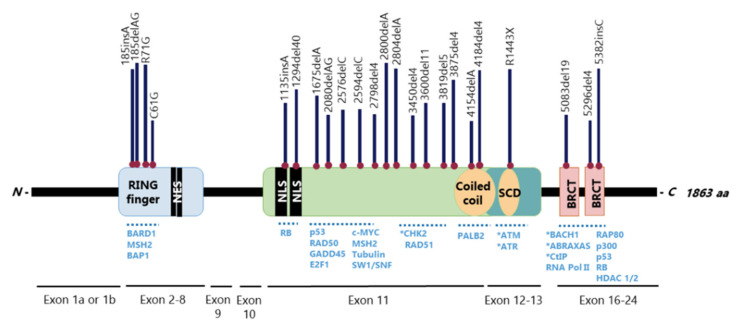
Structural organization of BRCA1 with respective interacting proteins and most prevalent mutations. Full length BRCA1 contains two conserved domains at its termini: *N*-terminus containing a really interesting new gene (RING) domain (exons 2–8) and tandem BRCA1 *C*-terminus (BRCT) repeats (exons 16–24). The BRCA1 RING domain interacts with BRCA1-associated RING domain (BARD1), mutS homolog 2 (MSH2) and the ubiquitin hydrolase BRCA1-associated protein 1 (BAP1). The BRCT domains form a phospho-binding module, recognizing a phospho-SPxF motif that allow BRCA1 A complex subunit (ABRAXAS), BRCA1-associated *C*-terminal helicase (BACH1) and *C*-terminal-binding interacting protein (CtIP) to physically interact with BRCA1. A number of other proteins may also bind to BRCA1 *C*-terminus, as p53, p300, receptor-associated protein 80 (RAP80), retinoblastoma (Rb), RNA polymerase II and histone deacetylases (HDAC1/2). Several proteins bind to exon 11, as Rb, E2F transcription factor 1 (E2F1), growth arrest and DNA damage-inducible 45 (GADD45), p53, checkpoint kinase 2 (Chk2), RAD51, SWItch/Sucrose non-fermentable (SWI/SNF), among others. The interaction of BRCA1 with partner and localizer of BRCA2 (PALB2) and BRCA2 is mediated by the coiled-coil domain. The serine cluster domain (SCD) contains multiple ataxia-telangiectasia mutated (ATM) and ataxia telangiectasia and Rad3-related protein (ATR) phosphorylation sites. BRCA1 contains two nuclear localization signals (NLS) and two nuclear export signals (NES). In the upper representation, the location and frequency of reported cases with BRCA1 pathogenic mutations are shown, including the most frequent C61G, 185delAG and 5382insC. (*) Phosphorylated proteins.

The *BRCA1* gene has 24 exons, two of them untranslated, and encodes a large 1863-amino acid phosphoprotein that harbours multiple functional domains, including the highly conserved *N*-terminal zinc-finger Really Interesting New Gene (RING) and two tandem BRCA1 *C*-terminus (BRCT) domains, in which mutations develop a tumorigenic potential [16,31] (Figure 2). BRCA1 nuclear-cytoplasmic shuttling is facilitated by nuclear localization (NLS) and nuclear export (NES) signals [16] (Figure 2). Over 60% of the *BRCA1* gene is composed by a centrally located exon 11, which encodes two NLS and binding sites for several proteins [32,33] (Figure 2). This is one of the largest human exons (encoding 1142 amino acids) that partially contributes to BRCA1 nuclear localization and activity on cell cycle regulation and DNA repair, being highly required for a functional HR [34,35]. Together with exons 12 and 13, exon 11 encodes a coiled-coil domain that mediates interactions with PALB2 and a serine cluster domain (SCD) that is phosphorylated by ATM and ATR [32,33]. Pathogenic mutations in exons 11-13 are frequently detected in breast and ovarian cancer patients, which reinforces the relevance of these exons in tumour suppression [32,33].

**Figure 3:**
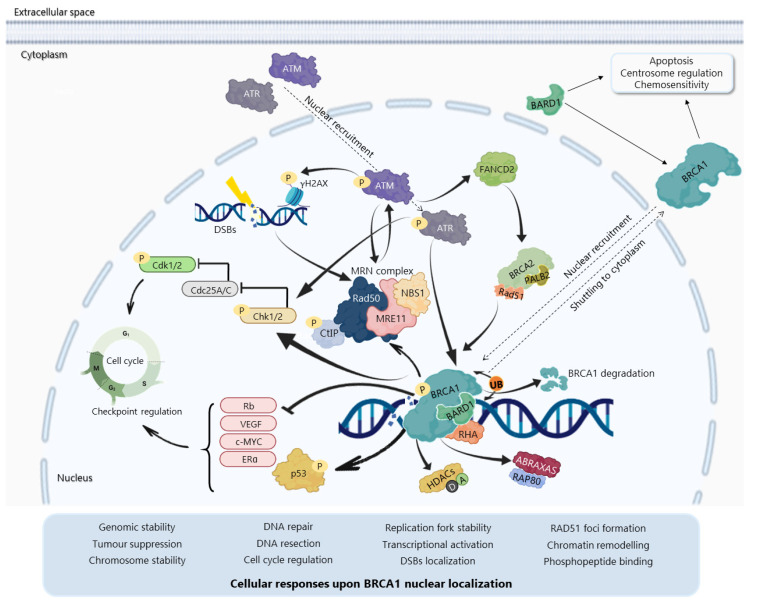
BRCA1 localization and molecular functions upon DNA damage. DNA damage activates the MRN complex (consisting of MRE11, meiotic recombination 11 homolog A; NBS1, Nijmegen breakage syndrome 1; and RAD50), which phosphorylates and recruits ataxia-telangiectasia mutated (ATM). Ataxia telangiectasia and Rad3-related (ATR) is also recruited to damaged sites during replication stress. DNA damage can also directly activate ATM/ATR, which can phosphorylate/activate several proteins as checkpoint kinases 1 and 2 (Chk1/2), histone H2AX (γH2AX) and BRCA1. Phosphorylated BRCA1 concentrates in focal areas of DNA damage. At nuclear foci, the BRCA1/BRCA1-associated RING domain (BARD1) heterodimer participates in several molecular mechanisms as DNA repair, cell cycle regulation and transcriptional activation, in association with protein binding partners as MRN complex proteins, *C*-terminal-binding interacting protein (CtIP), BRCA2/Partner and localizer of BRCA2 (PALB2), RAD51, BRCA1-associated *C*-terminal helicase (BACH1), BRCA1 A complex subunit (ABRAXAS), receptor-associated protein 80 (RAP80), histone deacetylases (HDACs), RNA helicase A (RHA), among others. The BRCA1-BARD1 heterodimer ubiquitinates several proteins, including BRCA1 and BARD1 although with no degradation by auto-ubiquitination, resulting in increased BRCA1 E3 ubiquitin ligase activity. BARD1 phosphorylation abolishes the heterodimer E3 ligase activity. BRCA1-BARD1 hetero-dimerization results in BRCA1 nuclear translocation and retention, while disruption of this complex leads to BRCA1 shuttling to cytoplasm, where BRCA1 influences apoptosis, chemosensitivity and centrosome regulation. Phosphorylation (P); Ubiquitination (Ub); Acetylation (A); Deacetylation (D); Cell division control protein 25 A/C (Cdc25A/C); Estrogen receptor (ER); Vascular endothelial growth factor (VEGF); Fanconi anemia group D2 (FANCD2); Cyclin-dependent kinase 1 and 2 (cdk1/2); Retinoblastoma (Rb).

The BRCA1 *N*-terminal RING domain dimerizes with BRCA1-associated RING domain (BARD1), forming stable heterodimers that enhance E3 ubiquitin-ligase activity and DDR [27,36,37,38,39,40] (Figure 3). BARD1 also plays a critical role in BRCA1 localization (Figure 3), since the BRCA1-BARD1 interaction masks NES of both proteins, resulting in BRCA1 nuclear translocation and retention [41,42,43,44]. In addition, BRCA1 can undergo proteolytic degradation upon disruption of the BRCA1-BARD1 heterodimer [45,46] (Figure 3). The specific function of the BRCA1-BARD1 heterodimer, its dissociation and interaction with other proteins might be regulated by post-translational modifications as phosphorylation and ubiquitination [36] (Figure 3). Thus, the BRCA1-BARD1 heterodimer plays a crucial role in tumour suppression, interacting with proteins involved in cell cycle, DNA repair, chromosome stability, chromatin modulation, replication fork stability, transcription, among others [47,48] (Figure 3). Although mutations in BARD1 do not affect the E3 ubiquitin ligase of the heterodimer [27,36,48], BRCA1 RING mutations affect its interaction with BARD1 and E3 ubiquitin ligase activity [18].

The BRCA1 BRCT domain functions as phosphopeptide recognition modules that enables the BRCA1 binding to phosphorylated partners as BRCA1 A complex subunit (ABRAXAS/ABRA1/CCDC98), BRCA1-associated *C*-terminal helicase 1 (BACH1/FANCJ/ BRIP1) and *C*-terminal binding interacting protein (CtIP/RBBP8) [16] (Figure 2 and Figure 3). Upon DNA damage, BRCA1 phosphorylation by ATM and ATR leads to post-translational modifications of BRCA1-binding proteins and to the subsequent activation of several associated proteins, including checkpoint kinases 1/2 (Chk1/2) and p53, which regulate cell cycle checkpoints [49] (Figure 3). Conversely, BRCA1 represses cell division control protein 25 A/C (Cdc25A/C) and c-Myc transcriptional activity and inhibits the expression levels of endogenous Estrogen Receptor (ER)α and vascular endothelial growth factor (VEGF) [50,51,52] (Figure 3). BRCA1 also regulates chromatin structure through acetylation and deacetylation of histone proteins by interaction with multiple histone deacetylases (HDAC1 and HDAC2) [50] (Figure 3). The BRCA1 interaction with RNA helicase A (RHA) also supports its role in the transcriptional machinery [53] (Figure 3). Thus, due to its rich functional domains, BRCA1 interacts with several transcriptional factors and numerous proteins encoded by tumour suppressors, oncogenes, DNA repair genes, cell cycle regulators, ubiquitin hydrolases and ligases, signalling transducers and chromatin modifying proteins (Figure 2), supporting the complex network involving BRCA1 [54,55].

### 3.1. BRCA1 as a Major Regulator of HR

**Figure 4:**
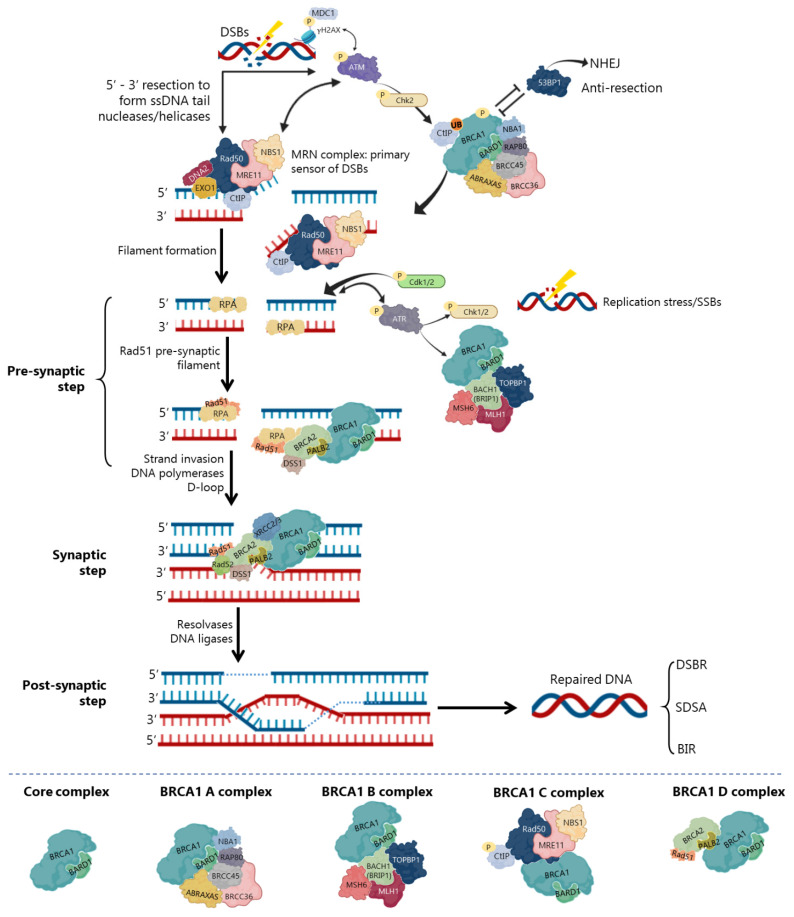
Double-strand breaks (DSBs) repair by homologous recombination (HR). DNA end resection occurs in the primary steps, a process that leads to nucleolytic degradation of DSBs 5′-ending strands to generate a 3′-end single stranded DNA (ssDNA). MRE11/RAD50/NSB1 (MRN) complex is the first to be recruited to DSBs sites, competing with Ku70/80 from non-homologous end joining (NHEJ) pathway. MRN complex phosphorylates ataxia-telangiectasia mutated (ATM) and recruits it to DSBs sites, leading to its auto-phosphorylation and phosphorylation of MRN complex. ATM phosphorylates checkpoint kinase 2 (Chk2) and the histone H2AX (γH2AX), recruiting the mediator of DNA damage checkpoint 1 (MDC1), which enhances ATM phosphorylation and promotes MRN and BRCA1 A complexes recruitment to damage sites. p53-binding protein 1 (53BP1) antagonizes BRCA1 in DSBs resection. Together with *C*-terminal-binding interacting protein (CtIP) (phosphorylated by MRN, ATM, cyclin-dependent kinase 2 (CDK2) and ubiquitinated by BRCA1), the MRN complex initiates DSBs resection to expose ssDNA with 3′ ends that undergo strand invasion into a homologous duplex (red), promoting HR. Ataxia telangiectasia and Rad3-related (ATR) is the primary sensor of replication stress (stalling of replication forks or formation of SSBs), which phosphorylation activates Chk1 and Chk2 and it is recruited to ssDNA sites. ssDNA tails are coated by replication protein A (RPA) followed by the formation of a D-loop structure through RAD51 load on the ssDNA. This is mediated by several proteins as BRCA2/Partner and localizer of BRCA2 (PALB2)/DSS1, BRCA1/BRCA1-associated RING domain (BARD1) and RAD51 cofactors, which allows RAD51 microfilaments formation and subsequent 3′-end strand invasion into the homologous DNA template and D-loop formation. The strand displaced by synthesis (red) anneals to the other resected end of the DSB (blue). To complete the HR process, the newly synthesized strand can dissociate to anneal to the other end. Different outcomes are possible, namely formation of Holliday junctions through DSBs repair (DSBR), synthesis-dependent strand annealing (SDSA) and break-induced DNA replication (BIR). Ubiquitination (UB); Phosphorylation (P); Exonuclease 1 (Exo1); DNA helicase/endonuclease 2 (DNA2); DNA topoisomerase 2-binding protein 1 (TOPBP1); BRCA1 A complex subunit (ABRAXAS); BRCA1-associated *C*-terminal helicase (BACH1); MutL homolog 1 (MLH1); MutS homolog 6 (MSH6); BRCA1/BRCA2-Containing Complex Subunit 36 (BRCC36) and BRCC45; Receptor-associated protein 80 (RAP80); X-Ray repair cross complementing 2 (XRCC2) and XRCC3.

In DSBs repair by HR, BRCA1 participates as a central component of macromolecular protein complexes, each one composed of unique protein binding partners, as phosphorylated ABRAXAS, BACH1 and CtIP [8,28,56,57] (Figure 4). These complexes, called BRCA1 A, B, C and D helped to recognize the multiple functions of BRCA1 not only in DDR, but also in the transcriptional regulation of genes involved in other cellular processes [56,58]. Interestingly, in all complexes, BRCA1 exists as a heterodimer with BARD1 (Figure 4), with distinct and sometimes overlapping roles for maintenance of genomic stability [56].

The repair of replication forks or DSBs is initiated by DNA strand resection to generate a 3′-tailed single stranded DNA (ssDNA) that will allow assembly of all HR machinery [59,60]. The MRE11/RAD50/NSB1 (MRN) complex is the primary sensor and co-activator of DSBs-induced cell cycle checkpoint signalling. It also functions as a repair effector of DSBs in both HR and NHEJ pathways [23] (Figure 4). In HR, the MRN complex forms a physical bridge, spanning the DSBs end to recruit/retain ATM at DSBs sites. This leads to its activation and autophosphorylation, along with phosphorylation of MRN complex by ATM [23,61,62] (Figure 4). ATM phosphorylates histone H2AX (γH2AX), which recruits the mediator of DNA damage checkpoint 1 (MDC1), enhancing ATM phosphorylation and promoting recruitment of MRN and BRCA1 A complexes to damage sites [62]. Chk2 is also phosphorylated by ATM to promote DNA end resection [63] (Figure 4). In the initial phase of HR, CtIP physically interacts with MRN complex (Figure 4), facilitating 5′ end-resection of DSBs. CtIP recruitment for DSBs ends and its phosphorylation is MRN-dependent, but still relies on ATM and cyclin-dependent kinase 2 (CDK2) phosphorylation, as well as ubiquitination by BRCA1 [58,62]. Although controversial, some studies have indicated that BRCA1-CtIP interaction may be dispensable for DNA mediated-resection. However, CtIP resection speed and length significantly decrease after disruption of the BRCA1-CtIP interaction [64,65]. Thus, BRCA1 C complex promotes DNA end-resection by regulation of MRE11–RAD50–NBS1–CtIP resection of nuclease complex, resulting in error-free HR events [32,33,47]. Subsequently, exonuclease 1 (Exo1) and DNA helicase/endonuclease 2 (DNA2) help BRCA1 C complex in DNA end-resection to generate stretches of 3′-ssDNA tails from the damaged DNA, which will be recognized/coated by the replication protein A (RPA) complex [62] (Figure 4).

Precluding the formation of secondary structures at the ssDNA, RPA occupies the 3′-tailed ssDNA derived from DNA end resection, protecting DNA tail from nucleolytic attack and removing the secondary structure [66]. Thus, in the pre-synaptic phase, RPA binds to the ssDNA tails activating ATR. Among its many functions, ATR activates Chk1 and Chk2 (leading to cell cycle delay to repair the damage) and BRCA1 [67] (Figure 4). Additionally, CDK1 and CDK2 appear to be associated with ATR recruitment and RPA phosphorylation. Despite not completely elucidated, this phosphorylation seems to promote the recruitment of DDR factors, as BRCA2/PALB2 and RAD51/RAD52, to the ssDNA [63]. To exchange RPA and facilitate RAD51 loading onto DNA, some mediators, like BRCA2 and PALB2, participate in RAD51-mediated pre-synaptic filament formation, a key intermediate that catalyses homologous pairing and initiates DNA strand invasion [24,33,47,66]. Meanwhile, BRCA2/PALB2 complex promotes RPA-RAD51 exchange on ssDNA and regulates RAD51 recombinase [61]. The BRCA1-BARD1 heterodimer helps RAD51-coated ssDNA to invade double stranded DNA (dsDNA) with homologous sequences, enhancing its ability to form a displacement loop structure (D-loop) [28,47]. BRCA1 and BRCA2 also interact during RAD51 recruitment to DSBs by PALB2 binding to BRCA1 coiled coil domain [68,69]. Some studies have shown that BRCA1 and BARD1 can also interact with RAD51, suggesting that these interactions are indispensable for HR and chromosome damage repair [47]. Accordingly, BARD1 mutations or deletions at residues 758–1,064 of BRCA1 (harbouring the RAD51-interaction domain) abolish the BRCA1-BARD1 ability to promote D-loop and synaptic complex formation [33,47], compromising HR activity and RAD51 nuclear localization [36,70].

Finally, with the support of DNA polymerases, the second end of the damaged chromosome is captured, and anneals to the complementary strand of the intact homologous DNA template [24,28]. At the end, the extended D-loop structure can be resolved by one of the three main mechanisms of DSBs repair (Figure 4). In the DSBs repair (DSBR), after priming DNA synthesis and sealing the break, the second end is captured, and a double Holliday junction intermediate is formed. After DNA synthesis and strands ligation, the two Holliday junctions can be resolved by the catalytic function of resolvases to generate crossover products, or dissolved to generate non-crossover products and complete the repair [24,71]. In the synthesis-dependent strand annealing (SDSA) model, the invading strand is displaced from a D-loop by helicase activity and annealed with the 3’ single-stranded tail to complete DNA synthesis and repair. Consequently, the intact chromosome has no risk to form a deleterious crossover product. Finally, the break-induced DNA replication (BIR) model is used when one of the DSBs ends is missing, leading to assembly of a partial replication fork that results in half crossover [71] (Figure 4). Neither HR nor NHEJ are fully dependent on the presence of an intact BRCA1, which suggests its supportive rather than indispensable function in these repair pathways. Actually, BRCA1-null cells still retain NHEJ activity [8,28]. However, NHEJ is frequently associated with increased error rates in cells during the DDR process.

### 3.2. BRCA1 Mutations and Tissue Specific Tumour Development

BRCA1^Mut^ carriers are at high risk for developing different types of cancer, including breast, ovarian, pancreatic, prostate, laryngeal and fallopian tube cancers [32,72,73]. Since its discovery, more than 1600 mutations have been identified in *BRCA1* [74], such as frameshift insertions/deletions, nonsense truncation mutations that lead to premature chain termination, and many single nucleotide polymorphisms in the coding or noncoding sequences. Over 70–80% of BRCA1 mutations result in dysfunctional or absent protein product. Also, a number of missense BRCA1 mutations present clinical relevance, being associated with increased risk of both hereditary and sporadic cancers [72,75]. However, the tumour aggressiveness, prognosis and therapeutic outcome vary with the type and location of the mutation that may occur in the RING and BRCT domains [16,31,72]. Many efforts have been developed to understand these clinical differences between BRCA1 mutations. Heterozygous BRCA1^Mut^ are commonly related to genetic deficiencies in other TSGs and DDR factors, such as phosphatase and tensin homolog (*PTEN*), *ATM/ATR*, *CHEK2* and *TP53* [16,58,72]. Accordingly, *TP53* mutations occur at higher frequencies in BRCA1^Mut^-associated cancers [76].

Some highly prevalent pathogenic BRCA1 mutations are more frequent in isolated groups (founder mutations), supporting the existence of distinct incidences among the world population. In particular, the BRCA1^Mut^:185delAG founder nonsense mutation is one of the most frequent in Asian, African, European and Ashkenazi Jews [58]. Although initially described with complete loss of BRCA1 expression, BRCA1^Mut^:185delAG alleles escape degradation, being translated from an alternative site downstream of the stop codon, which results in a RING-less protein [77]. Besides the loss of BRCA1-BARD1 interaction and subsequent E3 ligase activity, this alternative translation produces a stable and HR-proficient protein that retains the capability to interact with DNA and HR proteins [58,77]. Therefore, patients with BRCA1^Mut^:185delA display platinum and poly(ADP)-ribose polymerase (PARP) inhibitors (PARPi) resistance [58,77]. Interestingly, although located in the same domain as 185delAG, cells expressing BRCA1^Mut^:I26A produce a protein that lacks the E3 ubiquitin ligase activity, although retaining the heterodimerization with BARD1. Conversely, BRCA1^Mut^:185delAG presents a remarkable decrease in BARD1 binding ability [18].

The commonly detected pathogenic BRCA1^Mut^:5382insC is a frameshift mutation highly common in European countries, particularly in Ashkenazi Jewish descendants (Eastern European), Scandinavia and North Russia. Mutations at BRCT domains commonly present loss of protein expression associated with reduced transactivation activity, growth suppression [50] and aberrant cellular localization [78]. The BRCA1^Mut^:5382insC has a slightly truncated BRCT domain. Like 185delAG, also 5382insC was primarily described with a complete loss of protein. However, recent studies have shown that the truncated mRNA seems to encode a stable protein with potentially new cellular functions due to distinct protein-protein interactions [78]. Tumours that display homozygous BRCA1^Mut^:5382insC are associated with deficient HR activity and chemosensitivity [79]. However, the therapeutic response also depends on the tumour type. Indeed, although BRCA1^Mut^ at BRCT domain has been described as associated with chemosensitivity, the breast HCC1937 cancer cells displayed resistance to PARPi related to residual HR activity by retaining the integrity of RAD51 binding region [80].

Besides somatic mutations, *BRCA1* promoter hypermethylation and decreased *BRCA1* expression, by epigenetic silencing or gene depletion, can also render a dysfunctional BRCA1 pathway in non-hereditary cancers [16,81]. These mechanisms are described to likely contribute to the “BRCAness genotype”, associated with similar biological and clinical phenotypes to that of tumours harbouring BRCA1^Mut^. However, whether BRCAness mechanisms confer the same functional deficiency is still unclear [81].

A strong connection between triple-negative breast cancer (TNBC) and *BRCA1* status has been established. In fact, over 80% of BRCA1^Mut^ breast cancers are TNBC [82], which is an aggressive form of the disease. Although initially responsive to chemotherapy, most TNBC patients quickly relapse and acquire therapeutic resistance [83,84]. TNBC with pathogenic BRCA1^Mut^ also demonstrates to be particularly sensitive to platinum and PARPi agents, in both neoadjuvant and adjuvant settings [84].

Despite the lower prevalence, ovarian cancer is three-fold more deadly than breast cancer, with over 70% of patients having late-stage disease [85,86]. While type 1 ovarian cancer (low grade serous, mucinous, endometrioid and clear cell carcinoma and Brenner tumours) is commonly associated with mutations in genes like Kirsten rat sarcoma viral oncogene homolog (*KRAS*), phosphatase and tensin homolog (*PTEN)*, AT-rich interactive domain 1A (*ARID1A**)***, catenin beta 1 (*CTNNB1*), protein phosphatase 2 scaffold subunit A alpha (*PPP2R1A*) and phosphatidylinositol-4,5-bisphosphate 3-kinase catalytic subunit alpha (*PIK3CA*), type 2 (high-grade serous ovarian cancer (HGSOC), endometrioid and undifferentiated carcinomas) is associated with mutations in *BRCA1, BRCA2* and *TP53* [85,86]. In fact, over 10–15% of ovarian cancers are related to germline *BRCA1* and *BRCA2* mutations. Although still controversial, BRCA1^Mut^ carriers seem to have increased overall survival, likely due to their higher sensitivity to platinum-based therapy [75,87]. Also, these tumours have a dysfunctional DDR pathway that initially promotes sensitivity of cancer cells to chemo- and radiotherapy, although the cell population ultimately ends up developing therapeutic resistance [8]. Indeed, most BRCA1^Mut^-related ovarian cancer patients experience relapse associated with platinum resistance [88].

## 4. DDR Targeted Therapy for Cancer Treatment: The Synthetic Lethality Approach

**Figure 5:**
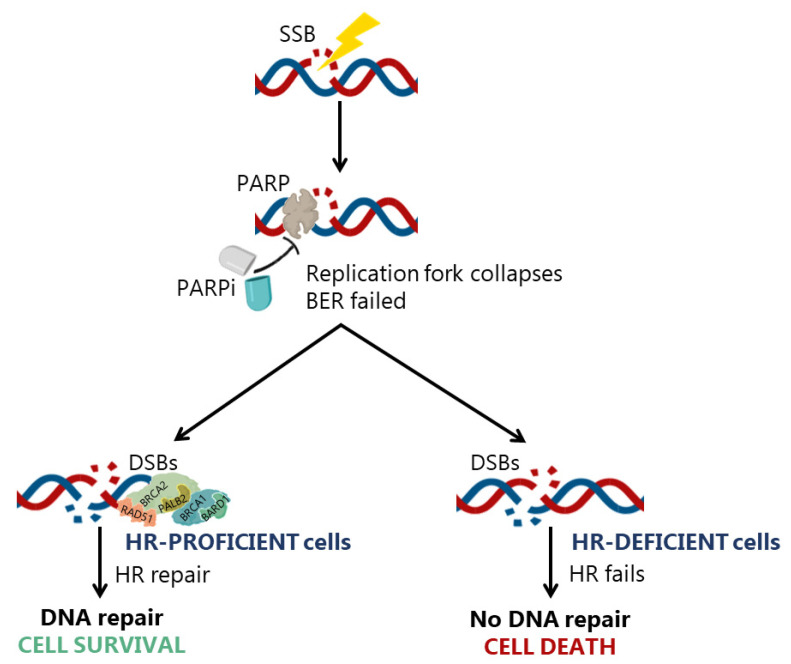
Underlying mechanism of synthetic lethality by poly(ADP)-ribose polymerase (PARP) inhibitors (PARPi). PARP enzyme is crucial in single-strand breaks (SSBs) repair. The pharmacological inhibition of PARP enzyme will assure that DNA lesions as SSBs will not be repaired by base excision repair (BER) mechanism and the damage will probably progress to double-strand breaks (DSBs). Homologous recombination (HR) is an important mechanism of DSBs repair. Therefore, the suppression of PARP enzyme through PARPi will produce distinct DNA repair responses by cells depending on their HR proficiency status. In HR-proficient cells, despite the failure of BER to repair SSBs, HR is able to repair the DSBs, contributing to the maintenance of genomic integrity and cell survival. Conversely, in HR-deficient cells, there are no reliable DNA repair mechanisms to repair the DNA damage, which leads to genomic instability and cell death.

A defective DNA repair pathway may sensitize cancer cells to chemo- and radiotherapy-induced cell death [22]. In particular, HR-deficient tumours, namely those harbouring deleterious BRCA1^Mut^, are highly sensitive to DSBs-inducing agents, such as interstrand crosslinking agents (e.g., platinum and alkylating agents) [21,87]. However, despite the initial encouraging response, these treatments tend to fail due to the development of resistance. To overcome this limitation, DDR targeted therapies have emerged as a promising strategy to be used as chemo- or radiosensitizers by exploiting defects in DDR pathways through the concept of synthetic lethality [89] (Figure 5). This approach relies on the presence of a specific gene product that resembles a phenotype induced by a mutation in cancer cells, compatible with viability, which combined with a second mutation in a different gene results in cell death. This strategy has allowed to enhance the selectivity towards cancer cells and to reduce the effective dose of conventional therapy, therefore minimizing side effects [89,90]. Over the last years, many drugs targeting DDR have been studied for potential use in cancer therapy.

### 4.1. PARPi

The concept of synthetic lethality gained clinical relevance in 2005, when BRCA^Mut^ patients were tested in clinical trials for their therapeutic response to the PARPi olaparib [91]. In fact, the pharmacological advantage of induced synthetic lethality has been extensively exploited during the last decades (Table 1 and Table 2), particularly with PARPi. In fact, the concept that a double-hit in DNA repair pathways results in synthetic lethality is the rationale for the use of PARPi in BRCA1^Mut^-related cancers [92] (Figure 5).

PARPi represent a family of nuclear enzymes involved in post-translational modifications of proteins and synthesis of poly(ADP-riboses) [92]. In particular, PARP1 and PARP2 have important roles in DDR of SSBs at base excision repair (BER) pathway (Figure 1). PARP also seems to facilitate HR by recruiting DNA repair factors as ATM and MRN complex to DSBs sites, also interfering with NHEJ by interaction with DNA protein kinase complex [92]. It is important to highlight the high selectivity of PARPi to HR-deficient cells. In fact, PARPi lack cytotoxicity on normal cells or cells with intact/residual HR function, since unrepaired SSBs are converted to DSBs and effectively repaired by HR [80,93].

Benzamide derivates were the first described PARPi, although they never entered clinical trials [94]. Later, olaparib (2014), niraparib (2017) and rucaparib (2018) were developed and approved by the U.S. Food and drug administration (FDA) for the treatment of advanced and chemoresistant ovarian cancers, in patients with germline BRCA1^Mut^ [20,95] (Table 1). In 2018, olaparib and talazoparib were approved for the treatment of metastatic and HER2-negative breast cancer, in patients who have already endure chemotherapy [96,97]. Interestingly, talazoparib showed to be over 100-fold more active at trapping PARP-1 and -2, with higher tumour selectivity and bioavailability when compared to PARPi such as rucaparib and niraparib [96]. Other PARPi were developed and entered clinical trials (Table 1), including veliparib [98], INO-1001 [99], pamiparib [100] and E7449 [101]. Recently, NMS-P118 was described as having excellent absorption, distribution, metabolism, and excretion profile and high in vivo efficacy in BRCA1^Mut^ TNBC, either as a single agent or in combination therapy [102]. Despite this, NMS-P118 has not yet entered clinical trials. PJ34 is also a potent PARPi able to improve the cytotoxic effect of chemotherapy on various tumour types, by inducing apoptosis and G2/M mitotic arrest. Thus, PJ34 may negatively impact cell growth in multiple ways in addition to PARP blockade to induce cell death in cancer cells [103]. Currently, several clinical trials with PARPi are still active, attempting to elucidate the efficacy and safety of these drugs (Table 1). However, despite promising and effective, many patients have shown severe side effects and heterogeneous response to PARPi, indicating the existence of complex inherent and acquired mechanisms of resistance [20]. Moreover, while 15% of ovarian cancer patients with BRCA1^Mut^ have five-years disease free survival upon treatment with olaparib, acquired resistance may occur within the first year of treatment by mechanisms as secondary frameshift mutations that restore the BRCA expression and HR function [20]. In fact, epigenetic changes in HR genes are a cornerstone for PARPi efficacy, with *BRCA1* and *RAD51C* methylation contributing to PARPi sensitivity, while demethylation has been associated with protein (re)expression and subsequent resistance [7,104]. Mitigation of replication stress, residual HR, PARP1 mutations, loss of 53BP1 function, overexpression of BRCA1 interacting partners, such as BRCA2, PALB2, DNA polymerase theta (POLQ), RAD51, RAD52, and drug-efflux pumps may also render cells more resistant to PARPi [20,80]. In HGSOC, overexpression of miR-622 also restores HR by downregulation of Ku complex, leading damaged cells to switch from NHEJ to HR [20]. Besides resistance to PARPi, these events may also lead to platinum resistance, although some exceptions have been reported regarding cellular resistance to PARPi, but not to platinum drugs [7,20,80,104]. Despite considerable advances with PARPi, much remains to be done, particularly considering that the resistance mechanisms are highly dependent on mutational profile of each tumour, its origin and prior treatments.

**Table 1:** Inhibitors of the DDR pathway that reached clinical trials.

	Cancer	Mechanism	Clinical Trial/Phase	Ref.
**Inhibitors of PARP**				
Olaparib (Lynparza; AZD-2281) Phase 4	Approved for BRCA^Mut^ metastatic BC (2018), advanced OC (2014), PC (2019) and prostate cancer (2020); advanced gastric and metastatic renal cell carcinoma	PARP1/2/3 inhibitor; binds within the nicotinamide-binding pocket in the ADP-ribosyl transferase catalytic site; synthetic lethality with HR defects, sensitizes cells to radiation and DNA damaging agents	NCT03344965/II NCT02184195/III NCT01924533/III NCT02810743/III NCT03286842/III NCT03786796/II NCT01874353/III	[104,105]
Rucaparib (AG-01499; Clovis) Phase 3	Approved for advanced OC (2016) and BRCA^Mut^ prostate cancer (2020); Solid tumours (e.g., PC and metastatic urothelial cancer)		NCT04171700/II NCT02975934/III NCT02042378/II NCT02678182/II NCT03533946/II NCT03413995/II	[20]
Niraparib (MK-4827; Zejula; Tesaro) Phase 3	Approved for recurrent OC (2017); BC, OC and PC with BRCA^Mut^; lung, head and neck cancer	PARP1/2 inhibitor; binds within the nicotinamide-binding pocket in the ADP-ribosyl transferase catalytic site, contacting with the regulatory subdomains; traps PARP to DNA damage sites; Talazoparib is effective in both BRCA^Mut^ and PTEN^Mut^ cancer cells	NCT01905592/III NCT03601923/II NCT03553004/II NCT03016338/II NCT03431350 I/II NCT03891615/I	[106,107]
Veliparib (ABT-888; Abbvie) Phase 3	Metastatic BRCA^Mut^ BC; NSCLC; HGSOC		NCT02163694/III NCT01149083/II NCT01657799/II NCT02890355/II NCT03044795/III NCT02158507/NA	[98]
Talazoparib (BMN 673) Phase 3	Approved for BRCA^Mut^ locally advanced or metastatic BC (2018); BRCA1^Mut^ OC; leukaemia		NCT02401347/II NCT02326844/II NCT02282345/II NCT02401347/II NCT03148795/II NCT03426254/I	[97]
INO-1001 Phase 2	Melanoma	Potent PARPi	NCT00272415/I	[99]
Pamiparib (BGB-290) Phase 3	Advanced solid tumours; OC; TNBC; prostate, brain and central nervous system tumours	Potent and selective inhibitor of PARP1 and PARP2	NCT03933761/II NCT03991494/II NCT03712930/II NCT04164199/III NCT03150862 I/II NCT03333915 I/II	[100]
E7449 (2x-121) Phase 2	Advanced OC; TNBC; metastatic BC; malignant solid tumours	Dual inhibitor of PARP1/2 (traps PARP1 onto damaged DNA sites) and tankyrase 1/2.	NCT03878849/II NCT01618136 I/II NCT03562832/II	[101]
**Inhibitors of ATM**				
M-3541	Solid tumours	Compete with ATP-binding site of ATM, inhibiting its catalytic function in DDR. KU-60019: downregulates pAKT reducing cell survival; combined with CDDP increases γH2AX and reduces RAD51 foci; specific for PTEN-deficient and p53^Mut^ cells upon IR. AZD-0156/AZD-1390: improved blood-brain barrier penetration	NCT03225105/I	[108]
AZD-0156	Advanced solid tumours		NCT02588105/I	[109]
AZD1390	Glioblastoma; brain neoplasms		NCT03215381/I NCT03423628/I	[110]
KU-60019	Kidney Cancer		NCT03571438/NA	[111]
**Dual inhibitors of PI3K/mTOR**				
NVP-BEZ235	Prostate cancer; advanced tumours including metastatic BC	Blocks ATM/ATR/DNA-PKs activity; induces chemo- and radio-sensitization, particularly in RAS-overexpressing tumours	NCT01717898I/II NCT01634061/I NCT01288092/II NCT01856101/II NCT01495247I/II	[112,113,114]
**Inhibitors of ATR**				
AZD6738 (ceralasertib)	Advanced solid tumours; lymphomas	Selective ATR inhibitor; phosphorylates Chk1 and increases γH2AX; promising with carboplatin or IR; antitumor activity in ATM-deficient xenograft models	NCT03770429/I NCT03682289/II NCT02630199/I NCT03462342/II NCT04298008/II NCT04361825/II	[22]
BAY1895344		ATR selective inhibitor	NCT04095273/I NCT03188965/I NCT04267939/I	[115]
VX-803 (M-4344)			NCT04149145/I NCT02278250/I	[115]
VX-970 (Berzosertib; M6620, VE-822)		Sensitizes PC and NSCLC cells to chemo- and radiotherapy; no toxicity in normal cells/tissues	NCT03718091/II NCT02487095 I/II NCT03641547/I NCT04052555/I NCT02157792/I NCT02627443 I/II	[116]
**Inhibitors of Chk1/Chk2**				
UCN-01	Advanced solid tumours	Block Chk1/2 activity by binding to ATP-binding pocket; induce cell cycle arrest in G1 (UCN-01) or G2 (MK8776) phases and apoptosis	NCT00082017/II NCT00072189/II NCT00045747/II NCT00072267/ II	[117]
GDC-0425			NCT01359696/I	[118]
MK-8776 (SCH900776)			NCT00779584/I NCT01521299/I NCT01870596/II NCT00907517/I	[119]
SRA-737			NCT02797964I/II NCT02797977I/II	
AZD7762		Inhibits Chk1/2 by interaction with their ATP-binding pocket; suppresses pCdc25C	NCT00937664/I NCT00413686/I NCT00473616/I	[120]
CBP-501		Inhibits kinases (MAPKAP-K2, C-TAK1, Chk1) that phosphorylate Cdc25C *Ser216*	NCT00551512/I NCT00942825/II NCT03113188/I NCT00700336I/II	[121]
Rabusertib (LY2603618)	Solid tumours (NSCLC; PC)	Chk1 selective inhibitor	NCT00415636/I NCT00839332I/II NCT00988858/II NCT01296568/I NCT01139775I/II	[119]
Prexasertib (LY-2606368)	SCLC, OC, TNBC, metastatic castrate-resistant PC	Dual Chk1/2 inhibitor	NCT01115790/I NCT02873975/II NCT02203513/II NCT02808650/I NCT02514603/I	
**Inhibitors of WEE-1**				
MK-1775 (AZD-1775)	NSCLC; advanced acute MM; OC; TNBC; PC; head and neck cancer; gastric cancer	Inhibitor of WEE1/2 and PLK1 kinases; potent in combination therapy	NCT01164995/II NCT02610075/I NCT01076400I/II NCT02087241/II NCT03012477/II	[122]
**DNA-PK inhibitors (NHEJ pathway)**				
LY-3023414	Prostate cancer and endometrial; NSCLC; TNBC; PC; lymphoma	Potent and selective ATP competitive inhibitor of class I PI3K isoforms, mTOR, and DNA-PK	NCT02549989/II NCT04032080/II NCT02575703/I NCT02549989/II NCT02443337/II	[123]
CC-122	Melanoma; advanced solid cancer; relapsed or refractory B-cell malignancies		NCT03834623/II NCT03310619I/II NCT02323906/I NCT02509039/I NCT01421524/I	[124,125,126]
CC-115	Glioblastoma; PC; Head and neck squamous cell carcinoma		NCT02833883/I NCT01353625/I NCT02977780/II	
M-3814 (MSC2490484A)	Various solid malignancies	DNA-PK-dependent inhibitor	NCT03116971/I NCT03724890/I NCT04172532I/II NCT04092270/I NCT02516813/I NCT02316197/I	[127,128]
AZD7648			NCT03907969I/II	[129]
VX-984 (M9831)			NCT02644278/I	[127,128]
**Dual inhibitors of HR and NHEJ**				
AsiDNA	Various solid malignancies	Prevents recruitment of repair enzymes (required for HR and NHEJ) at DSBs: acts as bait for DNA repair proteins or induces false DNA damage signalling	NCT03579628/I	[130]
**Inhibitors of RAD51 recombinase (HR pathway)**				
CYT-0851	B-Cell malignancies; advanced solid tumours	Reduces RAD51 migration to DNA damage sites	NCT03997968I/II	[131]

Clinical data obtained from clinicaltrials.gov (accessed on 14 May 2021); breast cancer (BC); triple-negative breast cancer (TNBC); ovarian cancer (OC); pancreatic cancer (PC); non-small cell lung cancer (NSCLC); small cell lung carcinoma (SCLC); myeloid leukaemia (MM); high grade serous ovarian carcinoma (HGSOC); ionizing radiation (IR); cisplatin (CDDP); phosphatidylinositol-4,5-bisphosphate 3-kinase (PI3K); mammalian target of rapamycin (mTOR); DNA-dependent protein kinase (DNA-PK); transforming growth factor-beta-activated kinase 1 (TAK1); polo-like kinase 1 (PLK1); not applicable (NA).

### 4.2. Other Inhibitors of the DDR Pathway 

Activation of cell cycle checkpoints is a critical step for DDR, giving cells time to repair. As such, inhibitors of key factors in cell cycle signalling sensitize cancer cells to radio- and chemotherapy [8,20,132]. Accordingly, inhibitors of phosphatidylinositol 3-kinase-related kinases (PIKK) family members, including ATM, ATR, and DNA-dependent protein kinase (DNA-PK), have been developed [8,20,132]. In 2004, the first selective inhibitor of ATM, KU-55933, was described [133]. However, its high lipophilicity has limited in vivo use [133]. Thereafter, KU-60019, a more effective analogue of KU-55933 with improved pharmacokinetic and bioavailability properties, was developed and is currently under clinical trials [111] (Table 1). CP-466722 is a potent, although reversible, inhibitor of ATM activity, which sensitizes cancer cells to the effect of ionizing radiation [134]. More recently, the ATM inhibitors AZ31, AZ32 [135], AZD0156 [109], and its improved version AZD1390 [110], were also developed, with the last two being tested in clinical trials (Table 1). In general, ATM inhibitors have shown promising results in combination regimens [136], although data regarding side effects are still not available.

Some ATR inhibitors have also been disclosed, being the naturally-occurring schisandrin B the first reported compound [137]. More effective compounds have followed the discovery of schisandrin B, namely NU-6027 [138], Torin 2 [139], and ETP-46464 [140]. However, despite their potent ATR inhibitory effect, they lack selectivity [140]. Likewise, NVP-BEZ235, currently under clinical trials (Table 1), was reported as a dual inhibitor of phosphatidylinositol-4,5-bisphosphate 3-kinase (PI3K) and mammalian target of rapamycin (mTOR) pathways, also inhibiting ATM/ATR and DNA-PKs, with considerable in vivo antitumor activity [112,113,114]. Later, a set of more selective and potent ATR inhibitors were described, including VE-821 [141] and the analogues VX-970 [116] and VX-803, BAY1895344 [115], AZ20 and AZD6738 [22]. The developed analogues revealed improved potency, solubility, bioavailability and pharmacokinetic properties compared to the counterparts [115]. VX-970, VX-804, BAY1895344 and AZD6738 are currently under clinical trials (Table 1), having AZD6738 and VX-970 undergone the greatest developments. 

Abrogation of the G2/M checkpoint by Chk1/2 and WEE-1 inhibitors is currently being tested in clinical trials (Table 1). Inhibitors of Chk1/2, downstream players of ATM and ATR, seem to act synergistically with agents that generate replication stress [119]. The first described Chk1 inhibitor was the staurosporine derivative UCN-01. However, the therapeutic application of UCN-01 has been hindered by its lack of specificity and long half-life, related to alpha-1-acid glycoprotein binding that leads to hyperglycemia [117,119]. Following UCN-01, several other Chk1/2 inhibitors have reached clinical trials (Table 1), namely CBP-501 [121], GDC-0425 [117], MK-8776, SRA-737 [119], AZD7766 [120], praxasertib, and LY2603618 [119]. Despite promising pre-clinical studies, results from clinical trials were not impressive, neither alone nor combined with other therapeutic agents. In fact, AZD7766, LY2603618 and MK-8776 not only have demonstrated modest efficacy, but also toxic side effects such as cardiotoxicity and thromboembolic events. As such, further clinical studies with these drugs were not pursued.

The WEE-1 inhibitor AZD-1775 potentiates the cytotoxic effect of several DNA-damaging drugs, also improving patients’ overall survival [122]. Several clinical trials are underway for a more rigorous selection of patients who may benefit from monotherapy or combination regimens with AZD-1775 (Table 1).

Upregulation of DNA-PK, a crucial component of NHEJ, has been observed in some cancers and, along with increased expression of Ku subunits, it is associated with radioresistance [20]. DNA-PK inhibitors have recently entered clinical trials, both as single agents and in combination therapy (Table 1). Based on the naturally-occurring flavonoid quercetin, DNA-PK-targeting inhibitors were developed, namely the non-specific Wortmannin [142] and LY294002, with high potency against DNA-PK, PI3K, polo-like kinase 1 (PLK1) and mTOR [143,144]. However, LY294002 proved to have unfavourable toxicological profile and poor stability, which precluded its clinical translation. Still, it led to the development of NU7026 and NU7441 (KU57788) [145], more potent and selective for DNA-PK [127]. Additionally, the compounds NU7427 [146], KU-0060648 [147] and NU5455 [148] were also effective against DNA-PK, sensitizing cells to radio- and chemotherapy-induced DNA damage [149]. Despite promising pre-clinical data, only few DNA-PK inhibitors have reached clinical trials, as LY3023414 [123,150], MSC2490484A, CC-122 and CC-115 [124,125,126], which target both DNA-PK and mTOR. VX-984 and M-3814 represent the latest generation of DNA-PK selective compounds that have proceeded into clinical trials (Table 1), improving radio- and chemotherapy efficacy [127,128]. However, despite well-tolerated as monotherapy, M3814 has shown side effects [127]. Finally, AZD7648 was described as a potent and highly specific DNA-PK inhibitor, having promising application in combination with standard therapies [129].

AsiDNA is a first-in-class DNA repair inhibitor that acts on enzymes involved in different DNA repair pathways, as HR, NHEJ, BER and SSA, providing an extensive DNA repair inhibitory activity rather than targeting specific DSBs proteins [130].

Small molecule inhibitors of MRN complex, RAD51, RAD52 and RAD54 have also been developed for targeting DSBs repair (Table 1 and Table 2). Regarding the MRN complex, the first compound identified was the MRE11 inhibitor 6-(4-hydroxyphenyl)-2-thioxo-2,3-dihydro-4(1*H*)-pyrimidinone (Mirin; Table 1), which targets MRE11 exonuclease activity, preventing ATM activation [151]. Mirin leads to HR failure and downregulation of NHEJ’s repair efficiency. Later, the Mirin analogues PFM39, PFM01 and PFM03, with selectivity towards Mre11 exo- (PFM39) or endonuclease (PFM01/03) activity, were also described [151].

One of the most promising approaches has been the simultaneous targeting of cancer cells with PARPi and other inhibitors of DNA repair factors, as RAD51 and RAD52, triggering a dual synthetic lethality, particularly in cancers with deficient DNA repair pathways [152]. Indeed, RAD51 overexpression has been described in several cancers, being associated with therapeutic resistance and poor prognosis [151]. Currently, RAD51 inhibitors able to interfere with RAD51 ssDNA-binding ability have been described, including DIDS [153], B02 [154], RI-1 and RI-2 [155,156], IBR2 and its more potent and specific analogue IBR120 [151] and the Chicago Skye Blue [157] (Table 2). An additional strategy has exploited the RAD51 overexpression in cancer cells using the compound RS-1, which stimulates DNA binding and genotoxic RAD51 recombination with subsequent induction of cell death [158]. Despite promising data in pre-clinical studies, none of these drugs have entered clinical trials since improvements in their solubility, toxicity and effectiveness are still needed. However, the new RAD51 inhibitor CYT-0851 [131] has recently reached clinical trials for safety, tolerability and pharmacokinetic studies (NCT03997968) (Table 1).

**Table 2:** DDR inhibitors under pre-clinical studies.

	Mechanism	Research Model	Ref.
**Inhibitors of RAD51 Recombinase (HR Pathway)**			
DIDS	Binds directly to RAD51; inhibits ssDNA- and dsDNA-binding, and joint molecule formation in DNA strand exchange assays; stimulates ATP hydrolysis; in vitro toxicity	In vitro DNA repair biochemical assays	[153]
B02	Inhibits RAD51-mediated ssDNA-binding activity; enhances cells sensitivity to IR, MMC, PARPi, doxorubicin and CDDP by inhibiting RAD51-dependent DSBs repair	In vitro DNA repair biochemical assays; mouse orthotopic xenograft of human TNBC	[154,159,160]
RI-1	Covalently binding to RAD51 protein, surface stably and irreversibly inhibiting its filament formation upon DNA damage; inhibit HR and disrupts DNA damage-induced RAD51 foci formation; sensitizes cancer cells to MMC	In vitro DNA repair biochemical assays; human embryonic kidney, ECC, BC and OS cell lines	[155,156]
RI-2			
IBR2 IBR120	Disrupt RAD51-binding to BRCA2 and RAD51 oligomerization; sensitize cancer cells to IR	In vitro DNA repair biochemical assays; BC xenograft model; imatinib-resistant T315I-Ba/F3 cells	[151]
Chicago Skye Blue (CSB)	Prevents RAD51 nucleoprotein filament formation by interfering with the RAD51 binding to ssDNA	In vitro DNA repair biochemical assays	[157]
**Inhibitors of RAD52 (HR pathway)**			
6-Hydroxy-d,l-dopa	Disrupts RAD52 oligomerization	In vitro DNA repair biochemical assays; BC and PC cell lines	[161]
D-103	Inhibit RAD52-mediated ssDNA annealing; tested in BRCA1^Mut^ and BRCA2^Mut^ cells	In vitro DNA repair biochemical assays; BC, OC, PC and OS cell lines	[162]
D-G23			
AICAR 5′-phosphate(ZMP)	Disrupts the RAD52-ssDNA interaction; targets intracellular RAD52; undergoes phosphorylation in the cytoplasm, preferentially killing BRCA1^Mut^ and BRCA2^Mut^ cells	In vitro DNA repair biochemical assays; BRCA1-deficient BCR-ABL1-32Dcl3 murine hematopoietic cells expressing GFP-RAD52; BC, PC and OS cell lines	[163]
(-)- EGC	Specifically bind to RAD52; disrupt the RAD52-ssDNA interaction and its annealing activity; kill BRCA2^Mut^ cells	In vitro DNA repair biochemical assays; human fibroblasts	[164]
NP-004255			
**Inhibitor of the BRCA1-BARD1 interaction (HR pathway)**			
BBIT20	Disrupts the BRCA1-BARD1 interaction	Co-immunoprecipitation and immunofluorescence assays; BC and OC cell lines; patient-derived cells and xenograft mouse models of OC	[165]
**Inhibitor of RAD54 DNA Branch Migration Activity (HR pathway)**			
Streptonigrin	Directly binds to RAD54 and inhibits its ATPase by reactive oxygen species generation	In vitro DNA repair biochemical assays	[166]
**Inhibitors of WRN DNA Helicase**			
NSC 19630	Specifically inhibits WRN helicase activity, but not its nuclease activity; increases cellular sensitivity to PARPi	In vitro DNA repair biochemical assays; human ECC, RC, CC, OC, BC and leukaemia cell lines	[167]
NSC 617145	Specifically inhibits WRN helicase activity, but not its nuclease activity; likely traps WRN on the DNA substrate	In vitro DNA repair biochemical assays; human ECC, OS and CC cell lines	[168]
**Inhibitor of BLM DNA Helicase**			
ML216	Inhibits helicase activity of BLM by disruption of its binding to DNA; inhibits WRN	In vitro DNA repair biochemical assays; BLM-complemented (PSNF5) and BLM-deficient (PSNG13) fibroblast cell line	[169]
**Inhibitors of MRE11 Endo- and Exonuclease**			
Mirin	Bind to active sites of MRE11, blocking DNA phosphate backbone rotation and inhibiting its exonuclease activity; inhibit MRN/DSBs-mediated ATM activation not affecting ATM protein kinase activity; G2/M-phase progression in HR-deficient cells	In vitro DNA repair biochemical assays; human OS cell line; human primary fibroblasts, NHEJ-deficient cells and FA cell lines	[170,171]
PFM39			
PFM01 PFM03	Bind near the dimer interface, blocking the ssDNA-binding path and disrupting endonuclease activity; enhance NHEJ while reducing the HR pathway (no DDR defects associated)	In vitro DNA repair biochemical assays; human primary fibroblasts, NHEJ-deficient cells and FA cell lines	[171]

Cisplatin (CDDP); ionizing radiation (IR); mitomycin C (MMC); triple-negative breast cancer (TNBC); wild-type (wt); werner syndrome helicase (WRN); bloom syndrome protein (BLM); breast cancer (BC); pancreatic cancer (PC); endocervical cancer (ECC); ovarian cancer (OC); osteosarcoma (OS); colon cancer (CC); renal cancer (RR); fanconi anemia (FA).

RAD52 inhibitors have also been developed to explore the synthetic lethality approach in cancers with BRCA^Mut^ or suppressed BRCA1-RAD51 pathway [152]. To date, the RAD52 inhibitors 6-hydroxy-d,l-dopa [161], 5-aminoimidazole-4-carboxamide ribonucleotide (AICAR) 5’-phosphate (ZMP) [163], D-103, D-G23 [162], and the naturally-occurring (−)-epigallocatechin (EGC [164]) and NP-00425 were described. However, none of them have reached clinical trials.

Considering that cells with truncation in BRCA1 *C*-terminus are more sensitive to DNA-damaging agents [58,72], the discovery of protein-protein inhibitors targeting the BRCT domain of BRCA1 also reveals to be an encouraging approach. Currently, only a small molecule-like [172] and phosphopeptides [173] were identified with the ability of targeting the BRCT domain of BRCA1. However, only the small molecule-like has in vitro activity due to its cell permeability [172].

Besides BRCA1^Mut^-related cancers, BRCA1^wt^ cancers with HR-deficiency due to impairment in other TSGs, such as p53^Mut^, might also benefit from DDR therapies. In fact, studies have unveiled that the poor prognosis and therapeutic resistance of p53^Mut^ tumours would be related to increased BRCA1^wt^ nuclear retention (associated with DNA repair and cell cycle checkpoints; Figure 3) [174]. Consistently, an interesting therapeutic approach for BRCA1^wt^ and p53^mut^ carriers would be the inhibition of the BRCA1-BARD1 interaction to improve the cellular response to DNA-damaging drugs. In fact, the disruption of the BRCA1-BARD1 interaction triggers the nuclear-to-cytoplasmic BRCA1 translocation and the subsequent depletion of BRCA1 HR activity [72]. Based on this premise, the BRCA1-BARD1 interaction inhibitor dregamine 5-bromopyridin-2-yl hydrazone (BBIT20) was recently identified by our group [165]. BBIT20 triggers DNA damage by promoting BRCA1 cytoplasmic localization and the subsequent reduction of major proteins involved in HR, in TNBC and ovarian cancer cells. The encouraging antitumor activity of BBIT20 in patient-derived cells and xenograft mouse models of ovarian cancer, particularly when compared to olaparib, may predict its great potential in precision therapy by targeting DDR [165].

## 5. Conclusions

Despite numerous studies with the aforementioned DDR inhibitors, only PARPi have been approved by the FDA for clinical use. Hence, the search for more effective agents targeting DDR pathways, in particular HR, remains of crucial relevance. In fact, the valuable application of DDR inhibitors in cancer treatment is undeniable. Particularly, the concept of synthetic lethality has gained significance in the field of DDR due to the multifactorial pathways that are deeply connected and that may compensate for each other, offering tumours an opportunity to develop drug resistance. In fact, synthetic lethality has emerged as a promising anticancer approach, more selective, efficient and lethal for malignant cells, and with less side effects when compared to conventional therapy. Nevertheless, its efficacy has also been greatly affected by the occurrence of resistance associated with the functional restitution of DNA repair pathways. On the other hand, it is well-accepted that their efficiency will depend on the correct identification of the genetic backgrounds for DDR deficiency. As such, the validation of biomarkers capable of stratifying the patients that may benefit from these therapies will be of high relevance to the success of these drugs. This will allow patients to be matched to the right treatment, driving the development of DDR targeted therapies for personalized cancer treatment.
